# OAGB 2025. A systematic review with meta-analysis of indications and results for primary procedures at 5+ years

**DOI:** 10.1007/s13304-025-02420-w

**Published:** 2025-10-02

**Authors:** Mario Musella, Sonja Chiappetta, Antonio Franzese, Pasquale Avella, Vincenzo Schiavone, Alessandra D’Ambrosio, Lucrezia Borrelli, Gerardo D’Amato

**Affiliations:** 1 Advanced Biomedical Sciences “Federico II” University, Naples, Italy; 2 Ospedale Evangelico “Villa Betania” – General Surgery, Naples, Italy

**Keywords:** OAGB, Long-term follow-up, Metabolic and bariatric surgery, Safety, Efficacy, Anemia, Hypoalbuminemia, Marginal ulcers, Bile reflux

## Abstract

One-anastomosis gastric bypass (OAGB) has gained increasing popularity as a metabolic and bariatric procedure due to its technical simplicity, promising weight loss and metabolic outcomes. However, its indications, long-term efficacy and long-term safety, remain the subject of an ongoing investigation. A systematic review of retrospective and prospective studies evaluating OAGB with a follow-up of minimum five years was conducted. 22 studies encompassing a total of 14,692 patients were included. The analysis included data on patient demographics, surgical indications, comorbidities, weight loss outcomes, and post-operative complications. Studies varied in design, with case numbers ranging from 101 to 2678 patients. Mean follow-up was 89.04 months (min 60 months, max 180 months). Patient age range was 33.8 to 47 years. Body mass index at surgery was between 33.4 and 54 kg/m^2^. Total weight loss rate ranged from 24.62% to 48.80% and excess weight loss percentage reached up to 94%, showing diabetes remission between 36.4% and 100%, hypertension resolution from 17% to 90.9%, and dyslipidemia improvement in up to 90% of cases. Long-term complications included anemia (1%), hypoalbuminemia (0.09%), marginal ulcers (0.19–7.7%), and bile reflux (0.8–9.8%). The necessity for revisional surgery was from 0.19 to 5.21%. OAGB appears to be an effective metabolic and bariatric procedure with high rates of weight loss and metabolic improvement in the long term. However, careful patient selection, nutritional monitoring, and long-term follow-up are essential to mitigate risks, such as anemia, hypoalbuminemia, marginal ulcers, and bile reflux. Further prospective, multicentric studies are warranted to establish standardized indications and optimize patient outcomes.

## Introduction

One-anastomosis gastric bypass (OAGB) is one of the three most commonly performed primary metabolic and bariatric procedures worldwide [1], and OAGB, as a revisional bariatric procedure, can be considered a safe and effective choice after primary restrictive procedures [2–4]. The International Federation for the Surgery of Obesity (IFSO) recognized OAGB as a bariatric/metabolic procedure in 2021 [5] and the American Society for Metabolic and Bariatric Surgery (ASMBS) endorsed the procedure in 2023 [6].

Scientific evidence regarding weight loss, and peri-operative and long-term complications is growing in the last decade; nevertheless, long-term outcome is warranted. Furthermore, patient selection and indication to OAGB is a point of discussion [7].

Weight loss outcome after OAGB is reported to be effective in the long term [8]. Two meta-analyses of randomized controlled trials confirm a significant reduction of HbA1c associated with a significant BMI reduction of OAGB [9] and a 28% higher 5-year remission rate of type 2 diabetes mellitus (T2DM) for OAGB compared to Roux-en-Y Gastric Bypass (RYGB) and sleeve gastrectomy (SG) [10].

On the other hand, long-term complications, such as bile reflux, malnutrition, vitamin D deficiency, iron deficiency, and marginal ulcers, are since the beginning a point of discussion [11].

Some studies have reported a higher incidence of clinical gastroesophageal reflux disease (GERD) and a greater need for conversion to RYGB following OAGB, particularly due to reflux-related complications, but also vitamin deficiencies and diarrhea. Conversely, other data suggest that the rate of revisional surgery after OAGB remains relatively low and acceptable over the mid- to long-term follow-up [12, 13].

This work aimed to analyze the current status regarding indications and long-term results of OAGB.

## Methods

This systematic review and meta-analysis included articles published until 30 April 2025, according to Preferred Reporting Items for Systematic Reviews and Meta-Analysis (PRISMA) guidelines [14], and it has been registered in the International Prospective Register for Systematic Review PROSPERO (registration number: CRD420251039169) [15]. The analysis of included articles quality was performed according to assessing the methodological quality of Systematic Reviews (AMSTAR) [16].

### Search strategy

A comprehensive literature search was carried out across three international databases (PubMed, EMBASE, and Cochrane) to find original English papers, focusing on the OAGB long-term outcomes.

Studies were selected using the following medical subject headings (MeSH): (OAGB OR one-anastomosis gastric bypass OR MGB OR mini gastric bypass OR SAGB OR single anastomosis gastric bypass) AND (indications OR results). Furthermore, the search strategy was flexibly modified based on the specific database requirements and further expanded by thoroughly identifying potential references from the screened texts.

An additional manual search using references of included studies was conducted. All reports obtained from the databases were exported to the reference management EndNote software (EndNote™ 21 version, Clarivate, Philadelphia, PA, USA).

The duplicated reports were managed according to Bramer et al*.* method [17].

All authors discussed and approved the search strategy, and the final review of the articles was carried out in May 2025.

### Inclusion and exclusion criteria

The main study objectives have been formulated using the Patients, Intervention, Comparison, and Outcomes (PICO) method [18].

Only articles written in English were selected to ensure accessibility and clarity in data interpretation.

We included studies involving adult patients (≥ 18 years old) undergoing OAGB as Metabolic and Bariatric Surgery intervention for severe obesity.

Eligible studies reported clinical outcomes related to weight loss, metabolic improvements, surgical complications, and post-operative quality of life (QoL). Both Randomized Clinical Trials (RCTs) and observational studies published in peer-reviewed journals will be considered, with no restrictions on geographic location or publication date. Studies must provide sufficient data on patient characteristics, surgical techniques, and follow-up duration to allow for comprehensive analysis.

RCTs, prospective and retrospective studies were included.

We reached out to the authors of reports that did not list pre-planned outcomes, to include eligible papers in our analysis.

Papers not published in English were excluded to maintain linguistic consistency and accessibility. Additionally, any articles without full-text availability were omitted to ensure a comprehensive evaluation of methodologies and results. Letters to the Editor were also excluded.

Studies with a follow-up period of less than 60 months were deemed insufficient for assessing long-term outcomes, while those involving fewer than 100 patients were excluded to enhance the reliability of the findings.

Descriptive articles lacking in analytical depth, systematic reviews, and meta-analysis were not considered.

These criteria collectively help refine the selection process, ensuring that only robust and relevant studies are included in the final analysis.

### Data extraction and quality evaluation

Five independent reviewers (P.A., V.S., A.F., L.B., and A.D.A.) evaluated studies through titles and abstract data. At least two blinded authors used Rayyan to identify and analyze relevant reports [19]. A third author resolved disagreements.

During the research process, the authors reviewed the bibliographies and citations of the eligible articles to identify any additional relevant reports that may have been missed in the initial literature search. Following this phase, we categorized the selected reports into three distinct groups, namely irrelevant, relevant, and unsure. Reports that were determined to be irrelevant by reviewers were excluded. Reports that were categorized as relevant or unsure by at least one reviewer were retrieved, and the full papers were evaluated.

All reports that meet the eligibility criteria were retrieved, and their full texts were carefully reviewed. Each reviewer then has selected a subset of reports to include, and these selected reports will be compared. Any discrepancies between the selected reports were discussed, and in cases where an agreement cannot be reached, a final decision will be made by another author (P.A.).

**Fig. 1:**
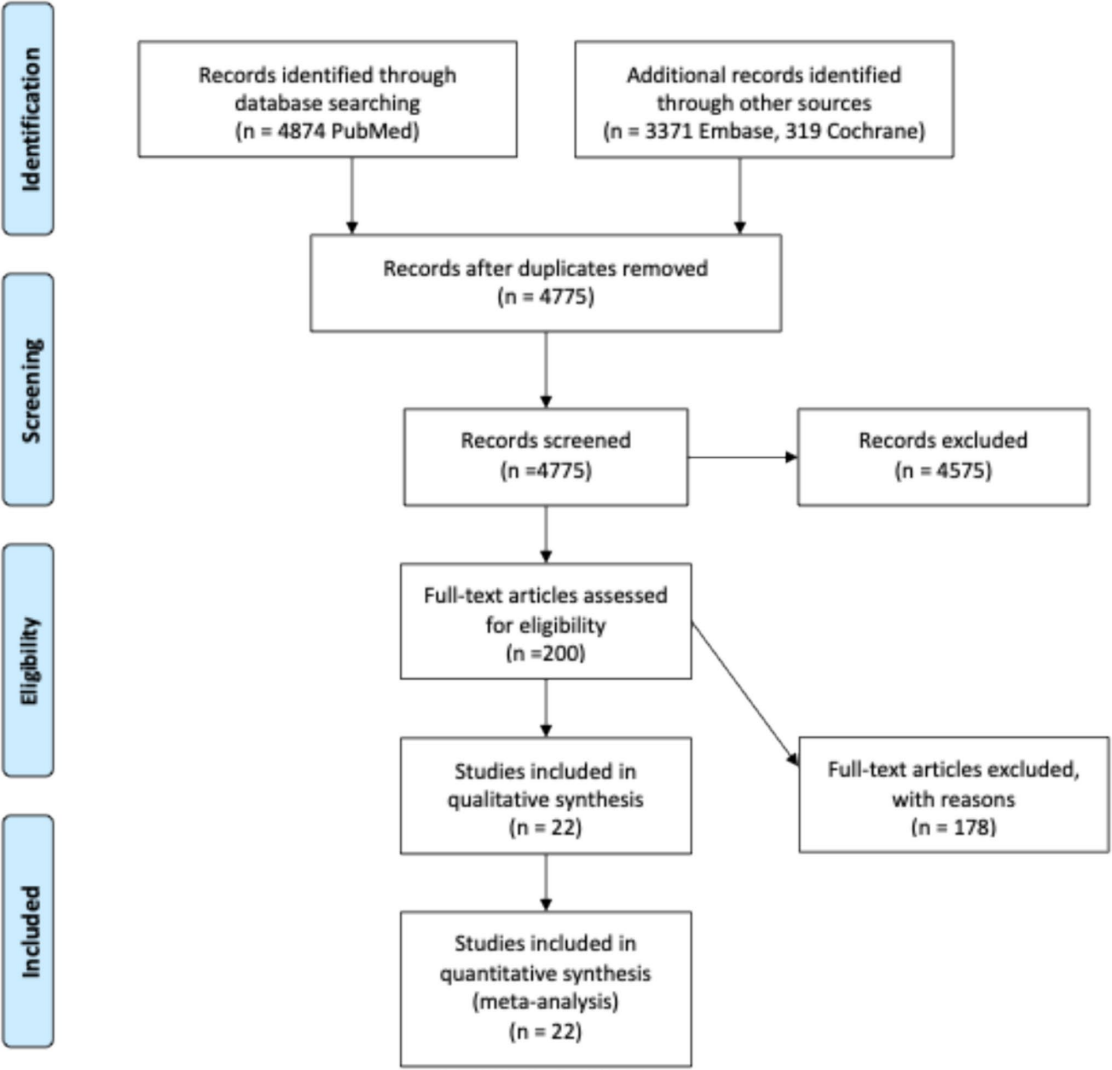
PRISMA Flowchart of our systematic analysis

The entire search and selection process was documented in a PRISMA flow chart (Fig. 1).

Using a standardized data extraction form, all authors recorded key details for each study, such as study name, name of the first author, year of publication, total number of patients, source from which study subjects were selected, study design, gender, median age, body mass index (BMI), comorbidity, peri-operative complications, BMI loss at follow-up, complications at last follow-up, and comorbidities remissions.

Only primary procedures were included in the meta-analysis.

Complete T2DM remission was defined as HbA1c < 6% in the absence of antidiabetic medications, complete GERD resolution according to absence of symptoms, and no medication use. Moreover, complete hypertension remissions were defined as blood pressure 140/90 or 120/80 mmHg in the absence of antihypertensive treatment, while dyslipidemia remission was achieved when patients presented with fasting plasma triglycerides < 200 mg/dL, total cholesterol < 200 mg/dL, and high-density lipoprotein cholesterol (HDL-C) ≥ 40 mg/dL, in the absence of pharmacotherapy.

Missing information was calculated based on the available data. At least two reviewers independently extracted data from all studies. Any discrepancies between reviewers in the data extraction were resolved by either consensus or a third reviewer.

Two reviewers performed the quality appraisal. A third reviewer assessed any disagreement.

### Ethical issues

Ethical approval or informed consent is not required for this systematic review and meta-analysis as it is based solely on previously published studies, without any direct contact with individual subjects.

## Results

### Patient demographics and baseline characteristics

This systematic review evaluates 22 studies [20–41] encompassing a total of 15,601 patients.

14,692 patients underwent primary OAGB. Patients undergoing OAGB in these studies were typically middle-aged adults and mean ages ranged from 33.8 to 47 years. Most cohorts had female patients. Preoperative BMI average ranged from 33.4 to 54 kg/m^2^. Four studies focused on super-obese individuals (BMI ≥ 50) [28, 32–34].

A high burden of obesity-related comorbid conditions was reported: Type 2 Diabetes (T2DM) was present in roughly 30–40% of patients on average and 40–50% of patients was affected by hypertension.

Obstructive sleep apnea (OSAS) and dyslipidemia were reported in about 20–40% of patients. Other obesity-related issues are listed in Table 1**.**

**Table 1:** Demographics

Authors	Year	Type of study (P, prospective; R, retrospective)	Number of cases	Age, years; mean-median	Female, *n*. (%)	Primary OAGB, *n*. (%)	Body Mass Index at primary surgery, kg/m^2^	Comorbidities at primary surgery				
								T2DM, (%)	Hypertensio*n*, (%)	OSAS, (%)	Dyslipidemia, (%)	Other, (%)
Kular et al. [20]	2014	R	1054	Mean ± SD 38.4 ± 9.6	712 (67.55)	1054 (100)	Mean ± SD 43.2 ± 7.4	63.94	71.91	32.35	61.95	
Kular et al. [21]	2016	R	128	Mean ± SD 41.6 ± 10.2	82 (64.06)	128 (100)	Mean ± SD 33.4 ± 3.3	NA	NA	NA	NA	
Musella et al. [22]	2017	R	2678	Mean ± SD 42.2 ± 3.8	1885 (70.38)	2251 (84.05)	Mean ± SD 45.39 ± 3.63	35.5	38.1	11.9	12	GERD 4.5
Carbajo et al. [23]	2017	R	1200	Mean ± SD 43	744 (62.00)	1173 (97.75)	Mean 46 (range 33–86)	15	32	93	56	
Alkhalifah et al. [24]	2018	R	1731	Mean ± SD 33.8 ± 10.4	1212 (70.01)	1731 (100)	Mean ± SD 40.4 ± 7.7	30.8	42.1	NA	54.6	
Baig et al. [25]	2019	R	1194	Mean ± SD 43.07 ± 11.42	646 (54.10)	1194 (100)	Mean ± SD 45.08 ± 8.82	NA	NA	NA	NA	
Liagre et al. [26]	2020	R	115	Mean ± SD 37.2 ± 11.7	101 (87.82)	97 (84.34)	Mean ± SD 43.2 ± 5.8	12.1	17.4	7.8	28.6	
Ruiz-Tovar et al. [27]	2020	R	300	NA	240 (80.00)	300 (100)	Mean ± SD 41.3 ± 8.2	35	43	NA	37	
Liagre et al. [28]	2021	R	245	Mean ± SD 39.7 ± 13.2	170 (69.38)	245 (100)	Mean ± SD 54 ± 4.9	17	30	29	17	
Soong et al. [29]	2021	R	498	Mean ± SD 32.1 ± 10.4	209 (41.96)	246 (49.39)	Mean ± SD 39.5 ± 7.9	47.6	NA	NA	NA	
Carandina et al. [30]	2021	R	385	Mean ± SD 43.2 ± 9.7	319 (82.85)	200 [52]	Mean ± SD 44.3 ± 6.7	84	28.1	13	NA	
Soong et al. [31]	2021	R	134	Mean ± SD 40.0 ± 12.9	89 (66.41)	134 (100)	Mean ± SD 39.5 ± 7.9	100	NA	NA	NA	
Kermansaravi et al. [32]	2022	P	197	Median 38 (18.2–69.3)	134 (68.02)	197 (100)	Median 53.7 (range 50–114.6)	17.8	16.8	20.8	24.4	GERD 12.1 Hypothyroidism 18.8 Urine stress incontinency 22.3
Jain et al. [33]	2022	R	63*	Mean ± SD 44.97 ± 9.73	38 (60.31)	63 (100)	Mean ± SD 39.73 ± 4.75	37	NA	NA	NA	
		R	38**	Mean ± SD 40.5 ± 10.97	24 (63.15)	38 (100)	Mean ± SD 51.92 ± 6.05	68	NA	NA	NA	
Plamper et al. [34]	2023	R	911	Mean ± SD 42 ± 11	694 (76.18)	911 (100)	Mean ± SD 50.97 ± 7.31	35	61.5	60.6	NA	
Hatami et al. [35]	2023	R	1381	Mean ± SD 39.34 ± 10.85	1081 (78.27)	1381 (100)	Mean ± SD 46.56 ± 6.5	23.5	20	12	40.4	
Jain et al. [36]	2024	PRCT	101	Mean ± SD 42.89 ± 14.02	62 (61.38)	101 (100)	Mean ± SD 44.32 ± 7.88	48.52	52.48	23.76	NA	
Rossoni et al. [37]	2024	R	114	Mean ± SD 47.0 ± 12.6	91 (79.80)	114 (100)	Mean ± SD 40.1 ± 5.6	19.1	43.6	NA	51.9	
van der Laan et al. [38]	2024	P	860	Mean ± SD 47[38–53]	647 (75.2)	860 (100)	Median 43 [40–46]	205 (23.8)	273(31.7)	85 (9.9)	107(12.4)	GERD 0.93
Makkapati et al. [39]	2024	R	152	Mean ± SD 41 ± 12.5	94 (75)	152 (100)	Mean ± SD 48 ± 20	46 (36)	42 (33)	55 (43)	63 (50)	GERD 11.18
Shahmiri et al. [40]	2024	R	1971	Mean ± SD 40.31 ± 11.21	1474 (74.8)	1971 (100)	Mean ± SD 46.68 ± 6.7	277 (14.1)	272 (13.8)	154 (7.8)	511 (25.9)	
Shahmiri et al. [41]	2025	R	151	Mean ± SD 39.40 ± 11.91	124 (82.1)	151 (100)	Mean ± SD 45.06 ± 6.66	13 (8.6)	17 (11.3)	5 (3.3)	25 (16.6)	

### Short-term surgical outcomes of OAGB

OAGB demonstrated a low 30-day mortality rate, ranging between 0.1% and 1%, primarily attributed to pulmonary embolism, anastomotic leaks leading to sepsis, and cardiovascular events in high-risk individuals. The overall early complication rate was estimated between 2% and 11.9%, with major post-operative morbidity (Clavien–Dindo grade III or higher) reported in 0.1% to 2.5% of cases. The most frequently encountered early complications included anastomotic leaks (0.15%–3%), gastrojejunal strictures (0.11%), and post-operative bleeding (0.8%–3%) (Table 2).

**Table 2:** Complications

Authors	Perioperative complications in the first 30 POD next to Clavien–Dindo, (%)	Revisional OAGB, (%)	Indication to redo-surgery	Perioperative complications in the first 30 POD next to Clavien–Dindo	Last follow-up in months	BMI at last follow-up, kg/m2; mean ± SD
Kular et al. [20]	- minor 4.6% - major 1.3%	NA	- NA	NA	72	26.2 ± 3.7
Kular et al. [21]	- staple line bleeding with shock (0.8%)	1.6	- Diabetic ketoacidosis 0.8%	NA	84	24.9 ± 2.4
Musella et al. [22]	- CD IIIA 0.3%, - CD IIIB 1.7%, - CD IV 0.03%, - CD V 0.1%	15.94	- Bile reflux 0.53%	NA	120	NA
Carbajo et al. [23]	- Major morbidity 2.7% - 30-day mortality 0.16%	2	- NA	NA	144	29.95
Alkhalifah et al. [24]	- 7.3%	4	- Malnutrition 2.48% - Anemia 1.38% - Protein malnutrition 0.80% - Intolerance 0.80% - Weight regains 0.51% - Bile reflux 0.34% - Excess weight loss 0.28% - Marginal ulcer 0.11% - Diarrhea 0.11% - Gastro-jejunal stricture 0.11% - Personal reason 0.11% - Dumping syndrome 0.05% - Titanium allergy 0.05% - Reflux esophagitis 0.05% - Gastro-gastric fistula 0.05% - Outlet obstruction 0.05%	NA	180	NA
Baig et al. [25]	NA	NA	- NA	NA	60	NA
Liagre et al. [26]	- Intra-abdominal abscess without documented leak 0.4% - pneumonia 0.4%	7.6	- Gastro-gastric fistula 4.12% -—Gastritis 2.06%	NA	96	28.3 ± 5.8 (19–50)
Ruiz-Tovar et al. [27]	NA	NA	- NA	NA	60	26 ± 4.9
Liagre et al. [28]	NA	4.48	- Internal hernia 2.4% - Bile reflux 1–2%	NA	80	NA
Soong et al. [29]	- CD I 2.24%, - CD II 11.94%, - CD IIIa 0%, - CD IIIb 1.49%,	6.9	- Malnutrition 5.3% - Marginal ulcer 0.8%, - Bile reflux 0.8%	NA	120	71.8% had BMI < 35
Carandina et al. [30]	- CD IIIb 2.59%	48	- Insufficient weight loss/weight regain 60%, - Slippage 13%, - Gastric wall erosion 2%, - Esophageal dilation 10%, - Port-related complications 15%	NA	180	31.3 ± 10.7
Soong et al. [31]	NA	NA	- NA	NA	60	27.6 ± 5.3
Kermansaravi et al. [32]	- Extra-luminal bleeding 3%, - Wound infections 1% - Mortality due to venous thromboembolism 1%	NA	- NA	NA	60	NA
Jain et al. [33]	NA	NA	- NA	NA	60	29.17 ± 2.05
	NA	NA	- NA	NA	60	32.88 ± 3.66
Plamper et al. [34]	- CD I 9.8% - CD II 2.0% - CDIII 2.6% - CD IV 1.6% - CD V 0.1%	9.8	- Bile reflux 2.6% - Weight regain/insufficient weight loss 2%	NA	60	NA
Hatami et al. [35]	NA	NA	- NA	NA	60	NA
Jain et al. [36]	NA	NA	- NA	NA	84 (64/101 pz)	31.36 ± 5.20
Rossoni et al. [37]	NA	NA	- NA	NA	60	NA
van der Laan et al. [38]	NA	NA	- NA	- Perioperative complication 2% - Gastrointestinal perforation 0.7% - Bleeding 0.8 - Liver injury 0.3% - Not classified 1(.1) Short-term complications 33 (3.8) Bleeding 3 (.3) Anastomotic leakage 3(.3) Intra-abdominal abscess 0 (.0) Intestinal obstruction 1 (.1) Intestinal injury 0 (.0) Other 6 (.7) Not classified 7 (.8)	60	29 (52)
Makkapati et al. [39]	NA	NA	NA	NA	120	± 6.4
Shahmiri et al. [40]	- Bleeding 2.23% - Anastomotic leak 0.15%	NA	NA	NA	108 FOR WEIGH 84 FOR COMORBIDITIES REMISSION	31.14 ± 5.60
Shahmiri et al. [41]	NA	151 (100)	NA	NA	60	NA

### Long-term surgical outcomes of OAGB

Mean follow-up of the included studies was 89.04 months (max 180 months).

**Fig. 2:**
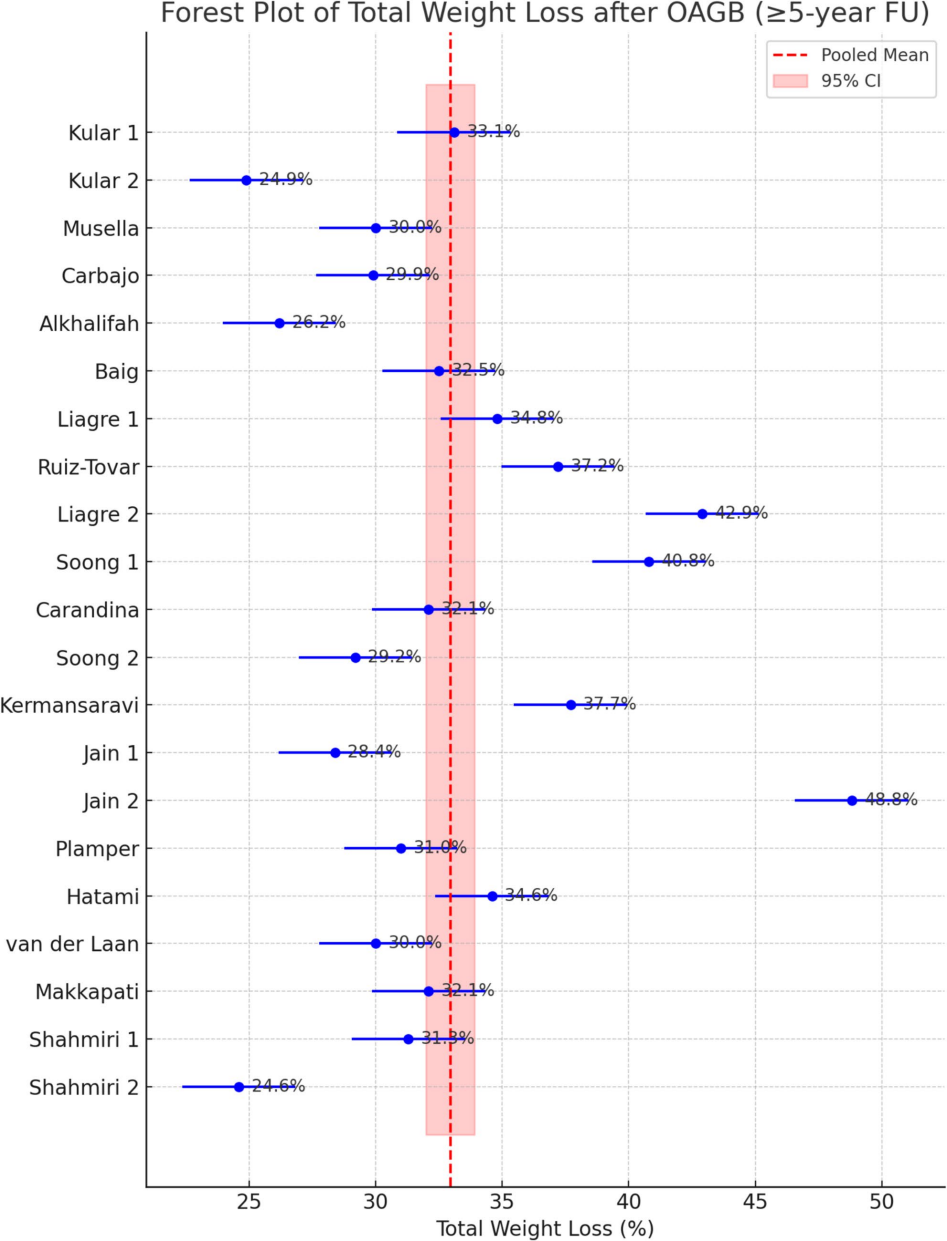
Forest Plot of Total Weight Loss after OAGB (≥ 5-year FU)

Long-term weight loss outcomes following OAGB were reported as %TWL and %TWL ranges between 23.22% and 48.8%, while %EWL varied from 56.31% to 94.0% at 5 + years. Using a random effects model, the pooled mean %TWL was 33.1% (95% CI 31.3–34.8%). Moreover, a high rate of heterogeneity was reported (*I*^2^ ≈ 98%). The funnel plot appeared symmetric and Egger’s test for small-study effects was non-significant (*p* = 0.89), indicating no significant bias in %TWL results (Fig. 2).

**Fig. 3:**
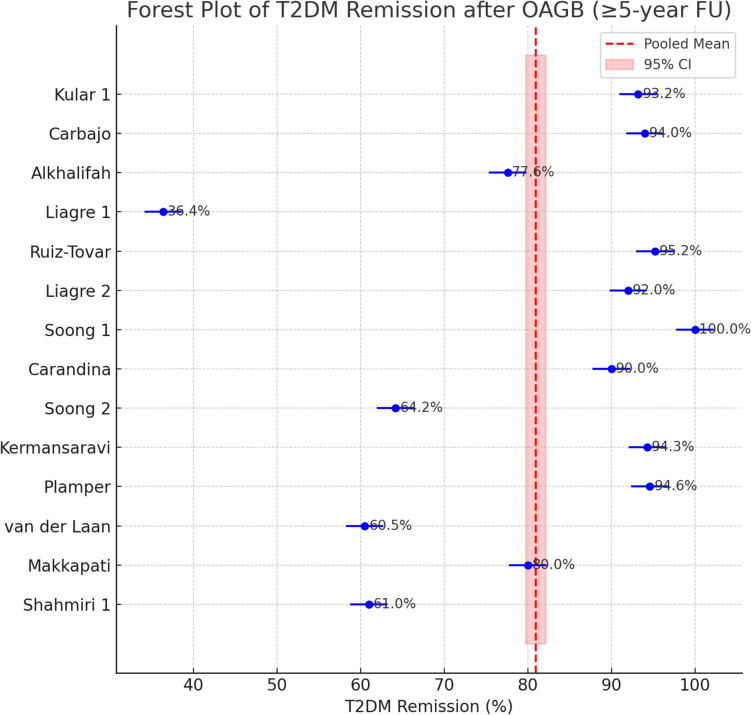
Forest Plot of T2DM Remission after OAGB (≥ 5-year FU)

The included studies showed improvement of obesity-related metabolic comorbidities. Diabetes remission rates ranged from 36.4% to 100%. The pooled T2DM remission rate (complete resolution of diabetes) was 86.4% (95% CI 78.5%–91.8%) of patients. Between-study heterogeneity was very high (*I*^2^ ≈ 96%). There was no sign of publication bias for the T2DM outcome. The funnel plot did not show asymmetry, and Egger’s regression test was not significant (*p* = 0.14), suggesting that the observed high remission rate is consistent across both large and small studies without selective reporting (Fig. 3).

**Fig. 4:**
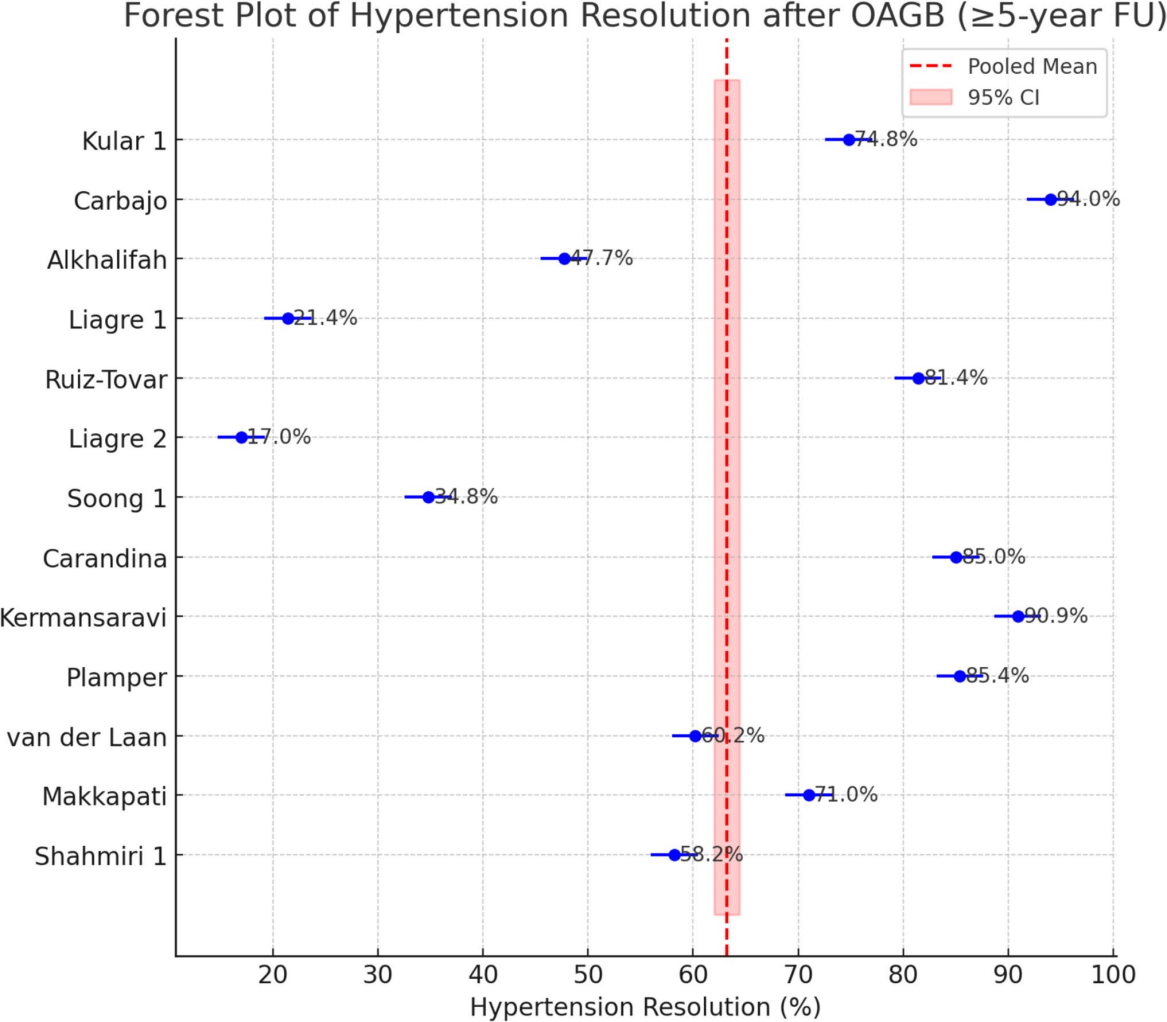
Forest Plot of Hypertension Resolution after OAGB (≥ 5-year FU)

The resolution of hypertension varied between 17% and 90.9%. The meta-analytic pooled hypertension resolution rate was 71.6% (95% CI 59.8%–81.0%). Heterogeneity was markedly high (*I*^2^ ≈ 97%), indicating that the proportion of patients with resolved hypertension differed greatly between studies. No significant publication bias was detected (Egger’s test *p* = 0.42) (Fig. 4).

**Fig. 5:**
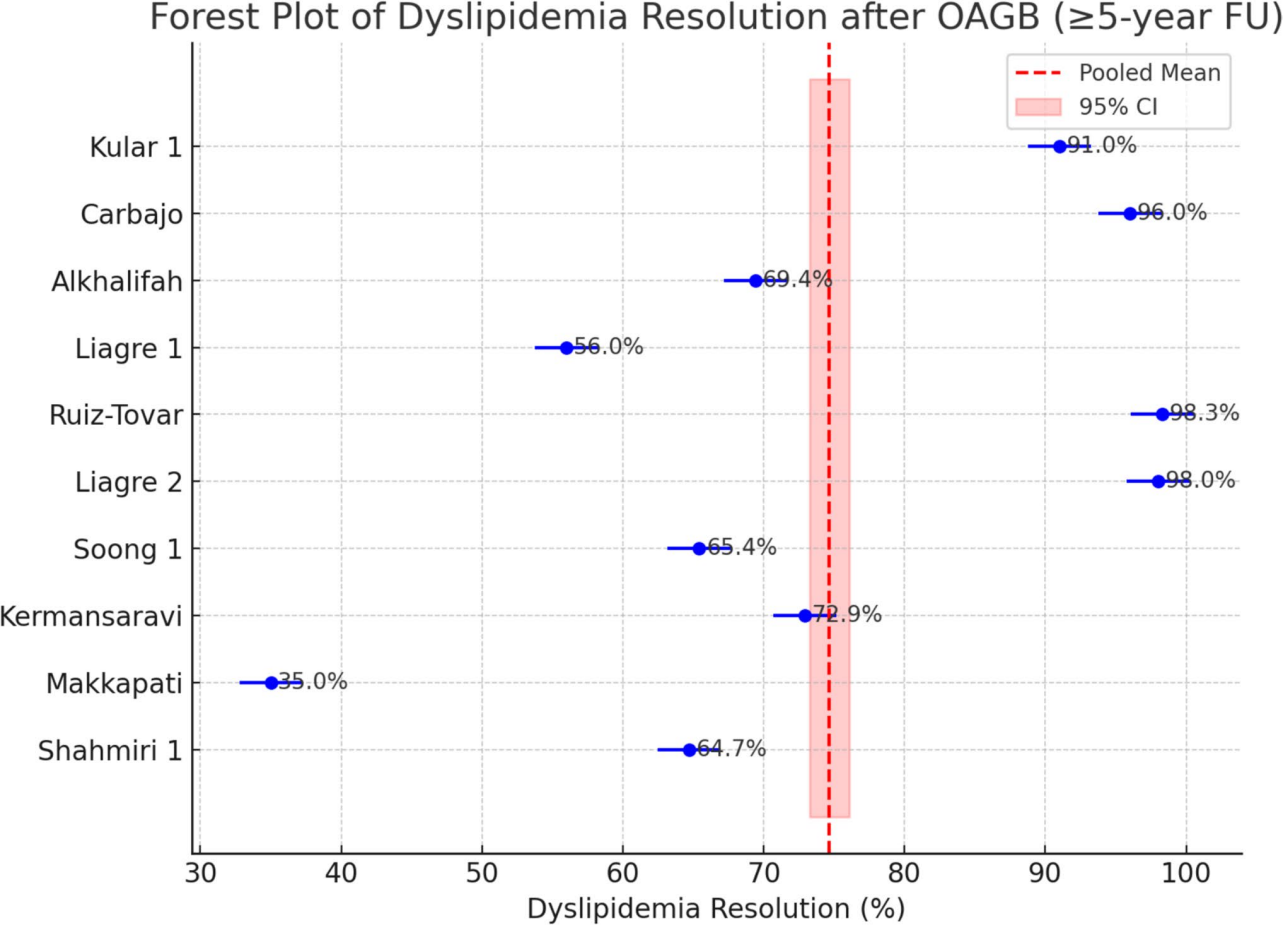
Forest Plot of Dyslipidemia Resolution after OAGB (≥ 5-year FU)

Dyslipidemia improvement was noted in 35% to 98.3% of cases. The meta-analysis found a pooled dyslipidemia resolution rate of 81.7% (95% CI 68.9%–90.0%). As with other outcomes, heterogeneity was extreme (*I*^2^ ≈ 97%). Despite this variability, no significant publication bias was evident. Egger’s test for dyslipidemia was non-significant (*p* = 0.43), and the funnel plot did not show a systematic skew, supporting the reliability of the pooled estimate (Fig. 5).

**Fig. 6:**
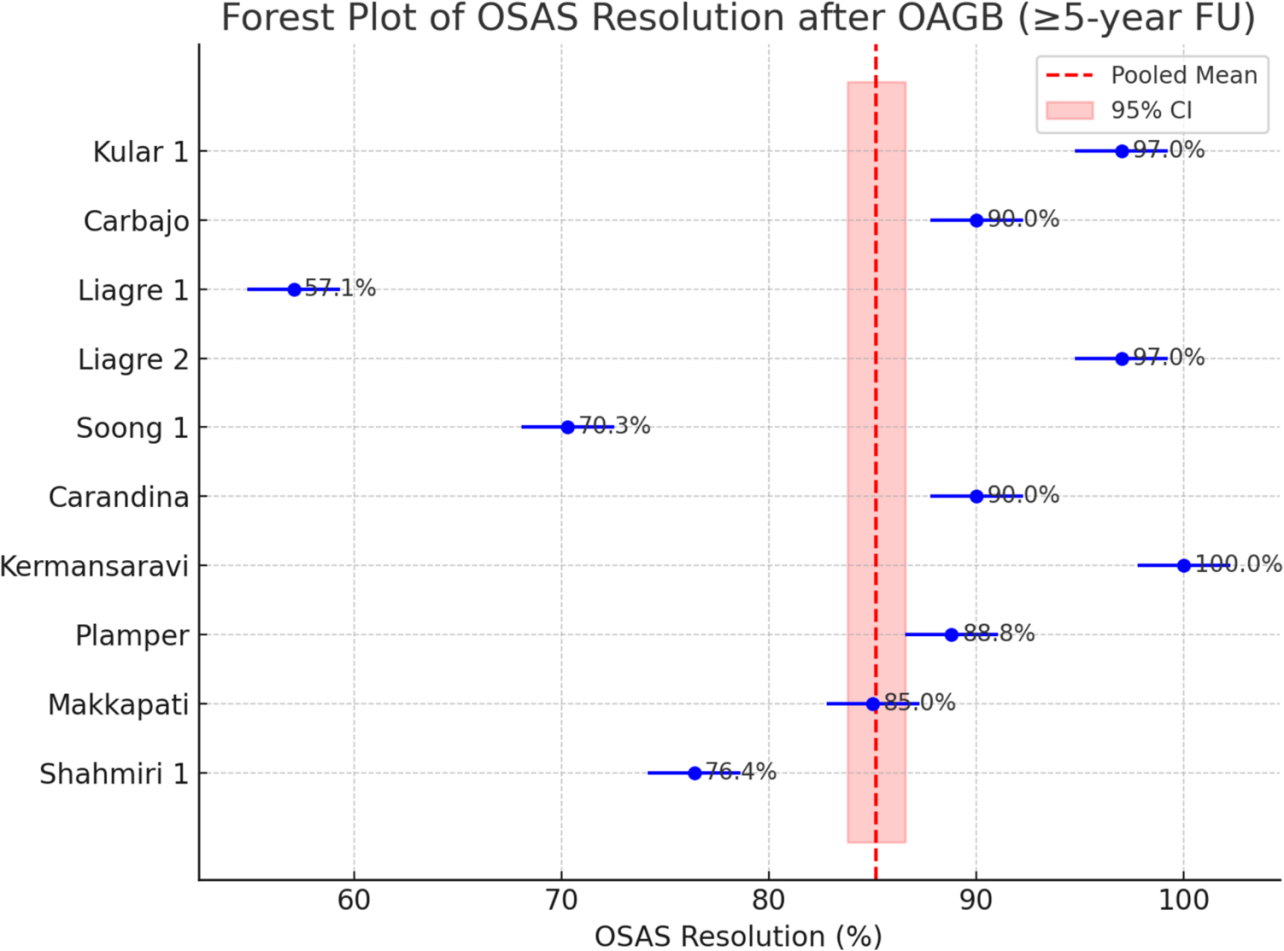
Forest Plot of OSAS Resolution after OAGB (≥ 5-year FU)

Additionally, the resolution of OSAS was achieved in up to 100% of patients who had undergone sleep studies pre- and postoperatively. The pooled OSAS resolution rate was 89.1% (95% CI 84.1%–92.7%). Heterogeneity was somewhat lower for OSAS than for other comorbidities but still substantial (*I*^2^ ≈ 85%, indicating significant between-study variation). Notably, a few studies achieved 100% OSAS remission, whereas others reported lower success rates around 60–70%. Funnel plot inspection did not reveal asymmetry for OSAS resolution, and Egger’s test was non-significant (*p* = 0.92), suggesting no publication bias and that the high pooled success rate is robust across studies (Fig. 6).

However, gastroesophageal reflux disease (GERD) outcomes following OAGB were more heterogeneous in patients with pre-existing GERD, with 48%–91.6% of patients experiencing symptomatic improvement, while 27% reported worsening reflux symptoms, sometimes necessitating conversion to RYGB.

OAGB was associated with several long-term nutritional and gastrointestinal complications. Malnutrition was reported in up to 34.3% of cases, necessitating close post-operative monitoring and life-long supplementation. Iron deficiency anemia affected 1% of patients. Furthermore, protein–energy malnutrition, characterized by hypoalbuminemia and sarcopenia, was seen in 0.09% of cases, requiring dietary modifications and, in severe cases, revisional surgery. Marginal ulcers were documented in 0.19%–7.7% of patients. Bile reflux was reported in 0.8–9.8% of the patients.

The rate of revisional surgery following OAGB ranged from 0.19% to 5.21%, with the most common indications being severe bile reflux (0.8%–9.8%), and marginal ulcer complications (0.19%–7.7%) **(**Table 3**).**

**Table 3:** Long-term outcomes

Authors	Total weight loss% at last follow-up; mean or median	Excess weight loss% at last follow-up, mean or median	Excess BMI loss (EBMIL) at last follow-up	Comorbidities resolution at last follow-up					Complications at last FU; %	Revisional surgery at last FU; %	Marginal ulcers at last FU; %	Bile reflux at last FU; %	Malnutrition at last FU; %
				T2DM, (%)	Hypertension, (%)	OSAS, (%)	Dyslipidemia, (%)	Other, (%)					
Kular et al. [20]	NA	Mean 85% at 6 years	NA	93.2	74.8	97	91	NA	- Low -n **0.09%** - Excess weight loss 0.09% - Anemia 6.45%	0.19%	0.19%	NA	NA
Kular et al. [21]	NA	78.5%	NA	NA	NA	NA	NA	NA	NA	NA	1.6%	0.8%	0.8%
Musella et al. [22]	NA	NA	NA	NA	NA	NA	NA	NA	NA	NA	1.1%	4.0%	0.7%
Carbajo et al. [23]	NA	70%	76.3	94	94	90	96	NA	- Stomal stenosis 0.5%	NA	0.5%	2%	1.1%
Alkhalifah et al. [24]	26.2%	70.3%	NA	77.6	47.7	NA	69.4	Hyperuricemia 59%	NA	NA	NA	NA	34.3%
Baig et al. [25]	32.53 ± 7.66%	72.96 ± 19.37%	29.40 ± 3.56	NA	NA	NA	NA	NA	NA	NA	NA	NA	NA
Liagre et al. [26]	34.8 ± 10.7%	84.8 ± 27.1 (0–154)	NA	36.4	21.4	57.1	56%	NA	- Bile reflux 27% - Hypoglycemic episodes 16.6% - Diarrhea 8.9% - Abdominal pain 5.1%	5.21%	4.3%	NA	NA
Ruiz-Tovar et al. [27]	37.2 ± 4.7%	94 ± 11%	15.3 ± 2	95.2	81.4	NA	98.3	NA	- Iron deficiency anemia 1%	NA	NA	NA	NA
Liagre et al. [28]	42.9 ± 11.8%	80.5 ± 21%	NA	92	17	97	98	NA	NA	NA	1.2%	NA	NA
Soong et al. [29]	40.8%	67.7%	NA	100	34.8	70.3	65.4	NA	NA	NA	0.8%	1.2%	5.3%
Carandina et al. [30]	32.1 ± 11.4%	- 62.9 ± 22.8: EWL > 75%: - 45 EWL 50–75%: 25% (n = 23). % EWL < 50%: 25% (n = 23)	68 ± 20.4	90	85	90	NA	NA	- Late complications 17.1%	NA	4.9%	9.8%	2.3%
Soong et al. [31]	29.2 + 10.6%	72.1(27.5)	NA	64.2	NA	NA	NA	NA	NA	NA	NA	NA	NA
Kermansaravi et al. [32]	37.7 (17.9–62.9)%	66.2 (29.3–98.9)	NA	94.29	90.9	100	72.9	GERD 91.6% Urine stress incontinency 93%	NA	NA	NA	NA	NA
Jain et al. [33]	28.40 ± 12.0%	n°33(80%)	NA	NA	NA	NA	NA	NA	NA	NA	NA	NA	NA
	48.80 ± 14.29%	n°30(90%)	NA	NA	NA	NA	NA	NA	NA	NA	NA	NA	NA
Plamper et al. [34]	NA	NA	NA	94.6	85.4	88.8	NA	NA	NA	1.53%	7.7%	8.3%	20.0%
Hatami et al. [35]	34.64 ± 8.37	77.60 ± 16.95	NA	NA	NA	NA	NA	NA	NA	NA	NA	NA	NA
Jain et al. [36]	27.71 ± 12.27 (64) 23.22 ± 12.66 (66)	59.99 ± 23.32	NA	91.18	84.2	62.5	NA	NA	NA	NA	NA	NA	NA
Rossoni et al. [37]	Na	Na	NA	NA	NA	NA	NA	NA	NA	NA	NA	NA	NA
van der Laan et al. [38]	30.0 ± 9.7%	74.0 ± 24.2	NA	Total remission 60.5 Partial remission 14.5	Total remission 60.2	NA	NA	NA	NA	NA	NA	NA	NA
Makkapati et al. [39]	32.1 ± 11.5%	82.4 ± 13%	NA	80	71	85	35	GERD 48%	46.8	NA	1.5%	NA	NA
Shahmiri et al. [40]	31.27 ± 9.85%	71.79 ± 23.20	NA	61.0	58.21	76.36	64.71	NA	NA	NA	NA	NA	NA
Shahmiri et al. [41]	24.62 ± 9.87%	56.31 ± 23.3	NA	NA	NA	NA	NA	NA	NA	NA	NA	NA	NA

The sensitivity analysis using the leave-one-out method for each of the 22 studies shows how the pooled means for total weight loss (%TWL) and comorbidity resolution outcomes (T2DM, hypertension, OSAS, and dyslipidemia) change when each individual study is excluded.

## Discussion

OAGB is known as a safe and efficient metabolic and bariatric procedure [5]. Analyzing 22 studies [20–41] and including 14,692 patients in this systematic review, we have seen that the cohort of patients undergoing OAGB is mainly female, aged 33.8–47 years and having a BMI of 33.4–54 kg/m^2^. It is well known that patients undergoing MBS are mainly female and middle-aged and that men, who undergo MBS, more often experience obesity-associated medical problems than women, and that they are more advanced in men [42]. Interestingly, the patient cohort of this systematic review shows a high burden of obesity-related comorbid conditions including T2DM in 30–40%, hypertension in 40–50%, and OSAS and dyslipidemia in about 20–40% of patients.

This means that indication for OAGB is quite always given in patients with obesity with ongoing illness [43]. In a recent performed Delphi consensus regarding patient selection in OAGB, there was a consensus that OAGB was a suitable option for patients with low BMI (30–35 kg/m^2^) with associated metabolic problems and in patients with BMIs more than 50 kg/m^2^ as a one-stage procedure. Both criteria are reflected as outliners also in this study [7].

Analyzing the long-term outcomes regarding OAGB, including only studies with a FU of more than 5 years, we have seen that %TWL ranged between 23.22% and 48.8% and %EWL varied from 56.31% to 94.0%. Comparing these numbers to the results of the SLEEVE-PASS trial at 5 years, which showed an estimated mean %EWL loss at 5 years of 49% (95% CI 45%–52%) after SG and 57% (95% CI 53%–61%) after RYGB (difference, 8.2 percentage units [44], it seems that %EWL after 5 years is much higher in patients undergoing OAGB. A recent performed network meta-analysis of randomized controlled trials comparing different surgery techniques with non-surgical therapy in diabetes patients, analyzing the primary endpoints HbA1c, BMI and diabetes remission, has shown that OAGB had a significant HbA1c reduction, which was associated with a significant reduction of BMI [9]. Furthermore, a recent systematic review and meta-analysis of randomized controlled trials has shown, that including 636 patients (*n* = 311 OAGB, *n* = 122 RYGB, *n* = 203 SG), OAGB showed a significant higher 5-year %EWL compared to the control group (*p* < 0.01) [10].

Comparing improvement of obesity-related metabolic comorbidities, the SLEEVE-PASS trial has shown at 5 years a complete or partial remission of type 2 diabetes in 37% after SG and in 45% after RYGB (*p* > 0.99). Medication for dyslipidemia was discontinued in 47% after SG and 60% after RYGB (*p* = 0.15) and for hypertension in 29% and 51% (*p* = 0.02), respectively [44].

The included studies of this systematic review showed much higher remission rates with a diabetes remission rates (defined as HbA1c < 6% without pharmacologic therapy) ranged from 36.4% to 100%, resolution of hypertension between 17% and 90.9%, and dyslipidemia improvement in 35% to 98.3% of cases.

Felsenreich et al. discussed the conversion rate of SG in the long-term and showed that amount to up to one-third of their cohort was converted [45], which is obviously a much higher conversion rate as reported in this systematic review with a rate of revisional surgery following OAGB which ranged from 0.19% to 5.21% in this group.

The three most feared complications of OAGB in the long-term are GERD and bile reflux, malnutrition, and marginal ulcers. The systematic review has shown that regarding GERD, 48% to 91.6% of patients experienced symptomatic improvement, while 27% reported worsening reflux symptoms and a prevalence of bile reflux of 0.8–9.8%. Also, the YOMEGA trial underlined twice the proportion of clinical GERD in the OAGB group than in the RYGB group (41% *vs* 18%), which seemed to worsen with time [12]. These findings underscore the importance of carefully assessing pre-operative GERD symptoms and the importance of performing upper endoscopy prior and post OAGB as indicated in the IFSO Endoscopy Position Statement [46]. Nevertheless, a recent systematic review has shown that GERD is higher after secondary OAGB (12%) than after primary OAGB (2%) [47]. Patients should be informed regarding this long-term complication in the pre-operative setting.

The rate of marginal ulcers in the long-term FU was 0.19% to 7.7% in this patient cohort and is quite in line with the prevalence of marginal ulcers after RYGB [48], which is described with a mean prevalence of 0.6–16% in a recent published ASMBS literature review [49]. The management of marginal ulcers might be the same as in RYGB with modification of risk factors and medical therapy focused on proton pump inhibitors. In case of complicated ulcers, surgical intervention is often required for the repair of the perforation or resection of the stricture [50]. Nevertheless, conversion from OAGB to RYGB due to marginal ulcer complications is described with 0.2%–5 in this patient cohort. These data underline the recently published data of a meta-analysis regarding marginal ulcers after OAGB, which describes that it is an uncommon complication following OAGB and that the majority of patients are treated conservatively with PPIs [51].

Finally, the risk of malnutrition is an important complication since it is correlated with important morbidity and mortality and life-long supplementation. On the one hand, we have to recognize 34% of patients with malnutrition in this cohort, and on the other side, the YOMEGA trial showed us that there is an improvement of the nutritional risk and diarrhea rate in the OAGB group at 5 years, even with a 200 cm bilio-pancreatic limb, and this suggests an intestinal adaptation with time that could contribute to lowering the late complication rate related to malabsorption [12]. Lifelong supplementation and regular FU are necessary to reduce the risk of malnutrition in our patients [52].

Finally, the findings suggest that while OAGB is effective for the majority of patients, a subset may require revisional procedures to mitigate complications or optimize long-term outcomes. Nevertheless, prevention including pre-operative upper endoscopy, modification of risk factors, regular FU, and life-long vitamin supplementation are necessary to reduce the risk of long-term complications.

### Limitations

The limitations of this systematic review include the presence of different and heterogeneous studies. Furthermore, half of the studies are retrospective studies. Third, the definition of remission of obesity-related metabolic comorbidities is reported differently in every study. Nevertheless, this is the first systematic review including 14,692 patients with a FU of 5 + years.

## Conclusion

OAGB is indicated in middle-aged patients in quite every BMI group with ongoing illness. OAGB has an important and durable weight loss and a significant improvement of obesity-related metabolic comorbidities in the long term (5 + years). Long-term complications, such as GERD, bile reflux, marginal ulcers, and malnutrition, are described and must be reduced by prevention. The rate of revisional surgery following OAGB is low in the long term.

## Data Availability

Data available on request from the authors.

## Abbreviations

MBS: Metabolic and bariatric surgery
OAGB: One-anastomosis gastric bypass
RYGB: Roux-en-Y Gastric Bypass
SG: Sleeve Gastrectomy
T2DM: Type 2 diabetes mellitus
GERD: Gastroesophageal reflux disease
FU: Follow-up
